# Dynamic Changes in the Phenotype of Dendritic Cells in the Uterus and Uterine Draining Lymph Nodes After Coitus

**DOI:** 10.3389/fimmu.2020.557720

**Published:** 2020-09-11

**Authors:** Ippei Yasuda, Tomoko Shima, Taiki Moriya, Ryoyo Ikebuchi, Yutaka Kusumoto, Akemi Ushijima, Akitoshi Nakashima, Michio Tomura, Shigeru Saito

**Affiliations:** ^1^Department of Obstetrics and Gynecology, University of Toyama, Toyama, Japan; ^2^Laboratory of Immunology, Faculty of Pharmacy, Osaka Ohtani University, Osaka, Japan; ^3^Research Fellow of Japan Society for the Promotion of Science, Tokyo, Japan

**Keywords:** feto-maternal tolerance, Kikume Green Red (KikGR), PD-L2, photoconvertible protein, tolerogenic dendritic cells, uterus

## Abstract

Dendritic cells (DCs) are essential for successful embryo implantation. However, the properties of uterine DCs (uDCs) during the implantation period are not well characterized. In this study, we investigated the dynamic changes in the uDC phenotypes during the period between coitus and implantation. In virgin mice, we evaluated the expressions of CD103 and XCR1, this is the first report to demonstrate uDCs expressing CD103 in XCR1^+^cDC1s and XCR1^+^cDC2s. On day 0.5 post coitus (pc), the number of uterine CD11c^+^CD103^–^MHC classII^high^CD86^high^–mature DCs rapidly increased and then decreased to non-pregnancy levels on days 1.5 and 2.5 pc. On day 3.5 pc just before implantation, the number of CD11c^+^CD103^+^MHC class II^dim^CD86^dim^–immature DCs increased in the uterus. The increase in mature uDCs on day 1.5 pc was observed in both allogeneic- and syngeneic mating, suggesting that sexual intercourse, or semen, play a role in this process. Meanwhile, the increase in immature uDCs on day 3.5 pc was only observed in allogeneic mating, suggesting that allo-antigens in the semen contribute to this process. Next, to understand the turnover and migration of uDCs, we monitored DC movement in the uterus and uterine draining lymph nodes (dLNs) using photoconvertible protein Kikume Green Red (KikGR) mice. On day 0.5 pc, uDCs were composed of equal numbers of remaining DCs and migratory DCs. However, on day 3.5 pc, uDCs were primarily composed of migratory DCs, suggesting that most of the uDCs migrate from the periphery just before implantation. Finally, we studied the expression of PD-L2—which induces immunoregulation—on DCs. On day 3.5 pc, PD-L2 was expressed on CD103^+^-mature and CD103^–^-mature DCs in the uterus. However, PD-L2 expression on CD103^–^-immature DCs and CD103^+^-immature DCs was very low. Furthermore, both remaining and migratory DCs in the uterus and uterus-derived-DCs in the dLNs on day 3.5 pc highly expressed PD-L2 on their surface. Therefore, our study findings provide a better understanding of the dynamic changes occurring in uterine DCs and dLNs in preparation for implantation following allogeneic- and syngeneic mating.

## Introduction

Dendritic cells (DCs) play an essential role in successful implantation and placentation in allogeneic- and syngeneic pregnancy (1–4). Moreover, uterus-resident DCs have been proposed to contribute to feto-maternal tolerance by regulating T cell activation (1, 5–8). Uterine DCs (uDCs) take up paternal antigens and present them to T cells in the draining lymph node (dLN), thereby inducing the regulatory T cells (Tregs) at the feto-maternal interface (5, 7, 9–13). The essential role of uDCs in the maintenance of feto-maternal tolerance during pregnancy has been examined. For instance, IDO-expressing DCs and plasmacytoid DCs (pDCs) act as tolerogenic DCs (tDCs) (4). These tDCs have been shown to possess the capability of immunoregulation by inducing Tregs, as well as T cell, anergy and deletion (6, 14, 15). However, few reports have examined the characteristics of uDC subsets in murine pregnancy (2, 4, 16–22).

Based on their morphological features and functions, DCs can be classified into conventional DCs (cDCs) and pDCs (4, 23–26). While each DC subset can present antigens to CD4^+^ T cells, CD103^+^ DCs can also present antigens to CD8^+^ T cells (23). Each DC subset is reported as tDC in food tolerance and tumor immunity (4, 15, 16, 27–31), however, little is known on the type of DC phenotype that increases in the uterus before implantation.

Transient inflammation in the uterine cervix and endometrium is observed after coitus-induced dynamic changes in immune cells (32–34). After insemination, neutrophils migrate rapidly into the uterus and are immediately decreased to non-pregnant levels by day 1.5 post coitus (pc) (35). Subsequently, DCs and macrophages migrate into the uterine endometrium to clear semen debris and make the uterus sterile (33). However, little is known about the phenotype, subsets, and spatiotemporal features of uDCs, as well as the migration of DCs between the uterus and the draining para-aortic lymph nodes. Hence, in the current study we aimed to investigate the dynamic changes in uDCs during allogenic- and syngeneic mating, from coitus to before implantation. To this end, we examined the surface markers of uDCs via flow cytometry, and migration of DCs using mouse line expressing photoconvertible fluorescent protein Kikume Green Red (KikGR) (36–38). We found that approximately 75% of uDCs were transformed to migratory DCs from day 2.5 to 3.5 pc. These migratory DCs may, therefore, play important roles in successful implantation.

## Results

### Uterine DCs Are Increased Following Coitus and Just Before Implantation

**FIGURE 1:**
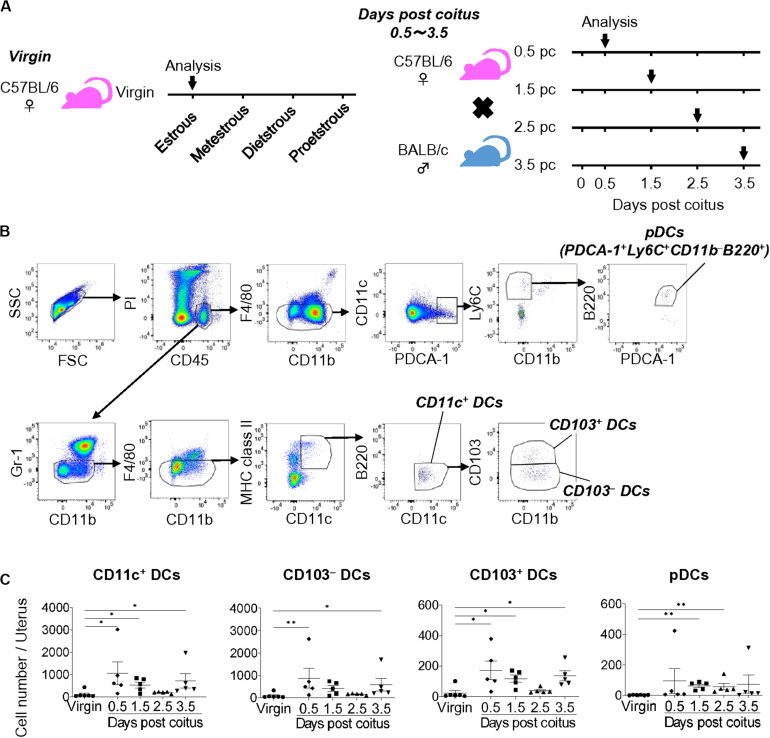
Cell numbers of uDCs after coitus in allogeneic mating. **(A)** Experimental time course. The DCs in the uterus in non-mated control virgin mice and mice on days 0.5, 1.5, 2.5, and 3.5 pc were analyzed by flow cytometry. **(B)** Gating strategy was used to identify CD103^–^ DCs, CD103^+^ DCs, and pDCs in the uterus. **(C)** Graphs show number of CD11c^+^ DCs and each DC subset in the uterus at each time point. A minimum of five samples from each time point were analyzed. Data represent mean ± SEM and are representative of three independent experiments. Statistical comparisons were performed using Kruskal-Wallis test with Dunn’s multiple comparisons test (^∗∗^*P* < 0.01, ^∗^*P* < 0.05).

To clarify the dynamic changes in uDC phenotype from coitus to implantation, we analyzed the time course for the classification of uDC subsets in allogeneic pregnancy (Figure 1A). Uterine DCs were identified as propidium iodide (PI)^–^ CD45^+^ Gr-1^–^ F4/80^–^ CD11c^+^ MHC class II^low–high^ B220^–^ cells (CD11c^+^ DCs) (Figure 1B and Supplementary Figure 1A). We then subdivided them into CD103^–^ CD11b^–/+^ (CD103^–^ DCs) and CD103^+^ CD11b^–/+^ (CD103^+^ DCs) cells (Figure 1B and Supplementary Figure 1B). Moreover, uterine pDCs were identified as PI^–^ CD45^+^ Gr-1^–^ F4/80^–^ CD11c^+^ PDCA-1^+^ CD11b^–^ Ly6C^+^ B220^+^ cells.

Compared to the non-mated control virgin mice, the total number of uDCs were increased on days 0.5 and 1.5 pc (9.7 and 4.9-folds, respectively), and returned to the non-pregnancy level on day 2.5 pc (1.7-fold), followed by an additional increase on day 3.5 pc (6.6-fold) (Figure 1C). The proportion of each DC subset in virgin mice showed that the majority of uDCs were CD103^–^ DCs (79.0%), followed by CD103^+^ DCs (17.8%), while pDCs (3.3%) were in minority (Supplementary Figure 1C). Hence the number of CD103^–^ DCs was similar to that of CD11c^+^ DCs (Figure 1C). Meanwhile, although the proportion of CD103^+^ DCs was smaller than CD103^–^ DCs, similar changes were observed in the time course (Figure 1C). Additionally, pDCs increased in number beginning on day 1.5 pc, however, continued to only represent a minor population (Figure 1C).

### Presence of CD103-Expressing uDCs in XCR1^+^-cDC1s and XCR1^–^-cDC2s

Recently, the characteristics of different DC subsets have been classified (26, 39). Therefore, here we sought to examine the expression of CD64, CD26, XCR1, and SIRPα to compare the presence of different DC subsets. First, we confirmed the exclusion of macrophages by staining for F4/80 and CD64 expression (Supplementary Figure 2A). From the total F4/80^+^ cell population, F4/80^+^ CD64^+^ cells accounted for 68%, while F4/80^+^ CD64^+^ cells accounted for 12.5% of the total CD64^+^ cell population, indicating that F4/80^+^ gating effectively excluded most of the macrophages (Supplementary Figure 2B). Next, we confirmed the proportion of DCs by examining CD26 expression (Supplementary Figures 2C,D). The expression of CD26 in CD11c^+^ DCs, CD103^–^ DCs, and CD103^+^ DCs was 69, 54.5, and 95.3%, respectively (Supplementary Figure 2D). These results indicate that PI^–^ CD45^+^ Gr-1^–^ F4/80^–^ CD11c^+^ MHC class II^+^ B220^–^ cells may be considered as DCs in the uterus. Moreover, although both CD103 and XCR1 have commonly served as markers of cDC1s, CD103 expression was also recently detected in cDC2s (26). Therefore, we further confirmed the DC subset be detecting XCR1 and SIRPα expression (Supplementary Figure 2E). In the CD11c^+^ DC population, the proportion of XCR1^+^ DCs and CD103^+^ DCs was 13.5 and 43%, respectively (Supplementary Figures 2E,F). Conversly, CD103 expression was detected in 13.1% of CD103^+^ XCR1^+^ DCs, and in 34.7% of CD103^+^ XCR1^–^ DCs (Supplementary Figures 2E,G), indicating the presence of CD103 in both cDC1s and cDC2s in the uterus.

### Mature DCs Increase After Coitus and Decrease to Non-pregnancy Levels Just Before Implantation

**FIGURE 2:**
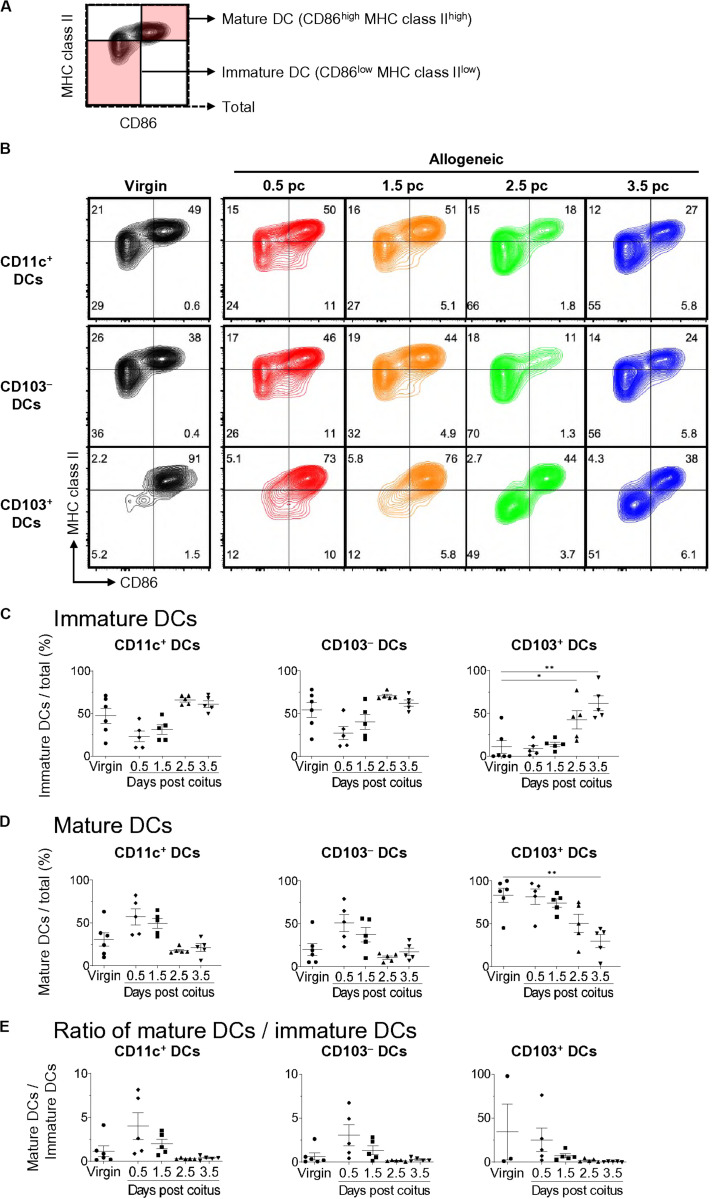
Time course for uterine immature and mature DCs after coitus in allogeneic mating. **(A,B)** Flow cytometry contour plots show immature DCs and mature DCs based on the expression of CD86 and MHC class II **(A)**, and representative plots in proportion to those within each DC subset at each time point **(B)**. **(C–E)** Graphs show the proportion of immature DCs **(C)** and mature DCs **(D)** out of the total DCs, and ratio of mature DCs/immature DCs **(E)** in each DC subset at each time point. A minimum of five samples from each time point were analyzed. Data represent mean ± SEM **(C–E)** and are representative of three independent experiments. Statistical comparisons were performed using the Kruskal-Wallis test with Dunn’s multiple comparisons test (^∗∗^*P* < 0.01, ^∗^*P* < 0.05).

We then subdivided each DC subset into CD86^low^ MHC class II^low^-immature DCs and CD86^high^ MHC class II^high^-mature DCs (Figure 2A) (15). The frequency of mature DCs among CD11c^+^-total DCs increased at days 0.5 and 1.5 pc compared to that in virgin mice, and then returned to the level observed in virgin mice on days 2.5 and 3.5 pc (Figures 2B,D,E). Meanwhile, the frequency of CD11c^+^-immature DCs increased on day 3.5 pc compared to that in virgin mice (Figures 2B,C,E). CD103^–^-mature DCs also increased at days 0.5 and 1.5 pc, however, the frequency of CD103^+^-mature DCs and pDCs did not change on days 0.5 and 1.5 pc. Hence, the increased number of mature uDCs on days 0.5 and 1.5 pc was likely due to increased CD103^–^ DCs. Moreover, the frequency of CD103^+^-immature uDCs on day 3.5 pc was significantly elevated compared to that in virgin mice, suggesting that an increase in immature uDCs on day 3.5 was due to increased CD103^+^ DCs.

### Characterization of uDCs Using t-Distributed Stochastic Neighbor Embedding (tSNE)

To define the specific DC subset present during the implantation period, we next analyzed the time course of uDC subsets via dimensionality reduction analysis, using tSNE. The pooled data for individual CD11c^+^ DCs and pDCs within the uterus across each time point (*n* = 26) was concatenated and visualized as a two-dimensional map by tSNE (Supplementary Figure 3). Results show that the clusters of each DC subset—particularly those of CD103^+^ DCs—were clearly divided into two clusters, MHC class II^high^ CD86^high^ and MHC class II^dim^ CD86^dim^ (Supplementary Figure 3A). The changes in distribution demonstrate (Supplementary Figures 3B,D,E) that the primary clusters in virgin mice consisted of cluster 6 made up of CD103^+^ mature DCs (Supplementary Figures 3A,C), clusters 8, 9, and 10 comprised of DCs without any specific surface markers, and cluster 12 made up of CD11b^+^ DCs. After coitus, the increased clusters on days 0.5 and 1.5 pc appeared as cluster 11 (Supplementary Figures 3D–F), which contained CD11c^+^ CD86^high^ MHC class II^high^ Ly6C^–^ PDCA-1^dim^ CD11b^+^ CD103^–^-mature DCs (Supplementary Figures 3A,C). Interestingly, new clusters on days 2.5 and 3.5 pc appeared as cluster 7 (Supplementary Figures 3D–F), containing CD11c^+^ CD86^dim^ MHC class II^dim^ Ly6C^–^ PDCA-1^dim^ CD11b^–^ CD103^+^-immature DCs (Supplementary Figures 3A,C). These results indicate that these newly appearing DCs before implantation are primarily CD103^+^ immature DCs.

### Differences in uDC Populations Between Allogeneic- and Syngeneic Mating

**FIGURE 3:**
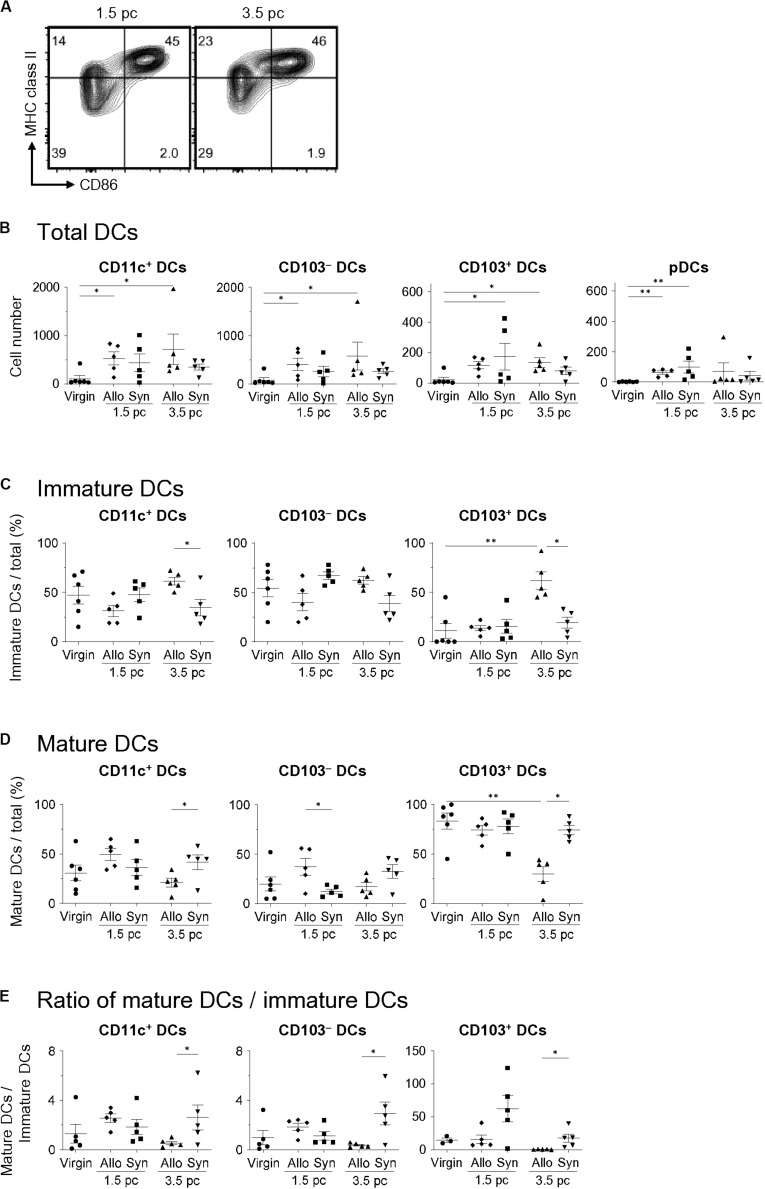
Comparison of uDC phenotype between allogeneic- and syngeneic mating. **(A)** Flow cytometry contour plots show immature DCs and mature DCs in syngeneic mating on days 1.5 and 3.5 pc. **(B,C)** Graphs show total DC number **(B)**, proportion of immature DCs **(C)** and mature DCs **(D)** out of the total DCs, and the ratio of mature DCs/immature DCs within each DC subset **(E)** in virgin, allogeneic-, and syngeneic mating mice at each time point. A minimum of five samples from each time point were analyzed. Data represent mean ± SEM **(B,C)** and are representative of three independent experiments. Statistical comparisons were performed using Mann-Whitney *U*-test (^∗∗^*P* < 0.01, ^∗^*P* < 0.05).

To examine the differences in the uDC populations in response to paternal antigens, we analyzed the characteristics of uDCs between virgin, allogeneic-, and syngeneic mating on days 1.5 and 3.5 pc (Figure 3A). The increase in uDCs on day 1.5 pc was observed in both allogeneic- and syngeneic mating (Figure 3B). There were no significant changes in the number or frequencies of uDCs between allogeneic- and syngeneic mating on day 1.5 pc (Figures 3B–E). However, an increase in CD103^–^- and CD103^+^- immature uDCs was observed in allogeneic mating, but not in syngeneic mating on day 3.5 pc (Figures 3C–E). These results suggest that the induction of immature DCs in the uterus before implantation is dependent on paternal antigens.

### Immature uDCs Are Increased Among the Infiltrating and Pre-existing DCs Just Before Implantation

**FIGURE 4:**
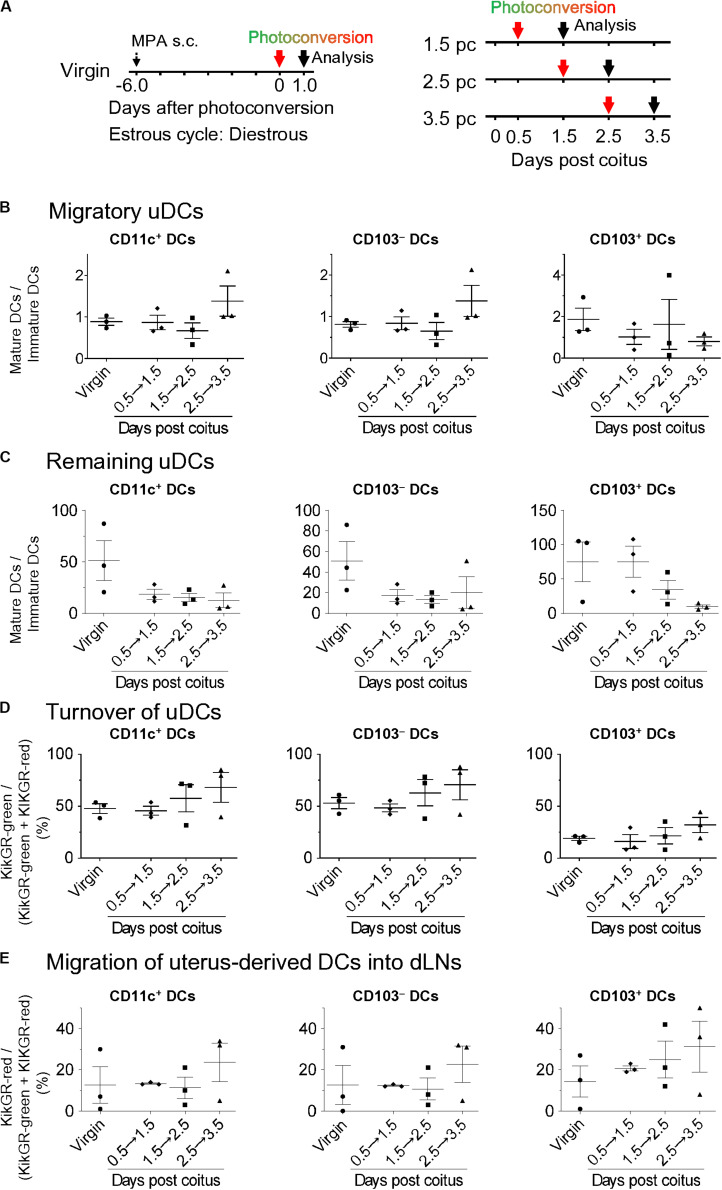
Time course for migratory DCs and remaining DCs in the uterus and dLNs after coitus in allogeneic mating. **(A)** Experimental time course. Virgin mice were synchronized at the diestrous stage, and the uteri of mice after coitus in allogeneic mating were photoconverted on days 0.5, 1.5, and 2.5 pc. The DCs in the uteri and dLNs were analyzed 24 h after photoconversion. **(B–E)** Graphs show the ratio of mature DCs/immature DCs **(B,C)** in each DC subset labeled with KikGR-green **(B)** and KikGR-red **(C)**, and proportion of each DC subset labeled with KikGR-green in the uterus, **(D)** and KikGR-red within MHC class II high DC subset in the dLNs **(E)** out of the total DCs 24 h after photoconversion. Three samples from each time point were analyzed. Data represent mean ± SEM **(B–E)** and are representative of three independent experiments. Statistical comparisons were performed using the Kruskal-Wallis test with Dunn’s multiple comparisons test.

Although the migration and pre-existence of uDCs using CFSE labeling in non-pregnant mice has been reported, little is known regarding how resident and migratory uDCs contribute to successful implantation (1). Thus, we elucidated the turnover of uDCs in KikGR mice (Figure 4A left). All DCs in the uterus were converted to KikGR-red immediately after photoconversion (Supplementary Figures 4A–D). Consequently, under such photoconversion conditions, we analyzed the KikGR-red remaining DCs and non-photoconverted KikGR-green migratory DCs at 24 h after photoconversion of the uterus with KikGR mice from day 0.5 to 1.5 pc, from 1.5 to 2.5 pc, and from 2.5 to 3.5 pc (Figure 4A right). This protocol allowed us to monitor the uDC turnover for 24 h on each gestational day. Results show that migratory DCs consisted of equal numbers of immature and mature DCs, with no change the proportion of immature/mature DCs in the migratory DCs over time (Supplementary Figures 5B, 6A,B and Figure 4B). In general, infiltrating DCs—which are of the immature phenotype—migrate to peripheral organs and are subsequently matured. Thus, equal proportions of mature and immature phenotypes in infiltrating DCs at 24 h from coitus to before implantation implies that the maturation rate of infiltrating DCs is not altered drastically. Alternatively, the remaining DCs were predominantly of the mature phenotype throughout the period after coitus, and just before implantation, with the proportion of mature DCs in the remaining DCs observed to gradually decrease (Supplementary Figures 5B, 6C and Figure 4C). During the analysis of changes due to cell turnover, the time course study revealed that the proportion of remaining DCs in the uterus gradually decreased by day 3.5 pc (Figure 4D). These results indicate that increased immature uDCs before implantation primarily make up the infiltrating DCs.

### DC Subsets of Uterine dLNs After Coitus to Just Before Implantation

It has been reported that paternal antigen-specific Tregs increase in the dLNs before implantation (13, 40). Uterus-derived DCs would then stimulate the paternal antigen-specific Tregs by presenting paternal antigens. Thus, it is important to understand dynamic changes in the migratory patterns of uDCs to dLN from coitus to before implantation. To this end, we analyzed the time course of MHC class II^high^ DCs in dLNs (Supplementary Figure 7A). Compared with virgin mice, the total number of migratory DCs in dLNs from day 0.5, 1.5, 2.5, and 3.5 pc were increased by 2.5, 14.4, 8.3, and 18.6-fold, respectively (Supplementary Figure 7B). In the changes of each DC subset, CD103^–^ DCs, CD103^+^ DCs, and pDCs levels were significantly increased on day 3.5 pc compared to those in virgin mice (Supplementary Figure 7B). Meanwhile, CD103^+^ DCs accounted for a minor population in dLNs throughout this period. To clarify the dynamic changes of migratory uDCs in the dLNs, we next analyzed the migration of uDCs using KikGR mice (Figure 4A). No KikGR-red DCs were detected in the dLNs immediately after photoconversion, indicating that photoconversion was restricted to the uterus (Supplementary Figures 4C,D). However, 24 h after photoconversion, we detected KikGR-red DCs in migratory DCs (CD11c^+^ MHC class II^high^) (Figure 4E), but not in lymph node DCs (LNDCs) (CD11c^+^ MHC class II^int^) (Supplementary Figure 7C), suggesting that uterine migratory DCs were exclusively CD11c^+^ MHC class II^high^. Furthermore, the time course study revealed that the migration of KikGR-red uterus-derived total DCs, CD103^–^ DCs, and CD103^+^ DCs in the dLNs showed an increasing trend by day 3.5 pc (Figure 4E).

### PD-L2^+^ Expression on DCs

**FIGURE 5:**
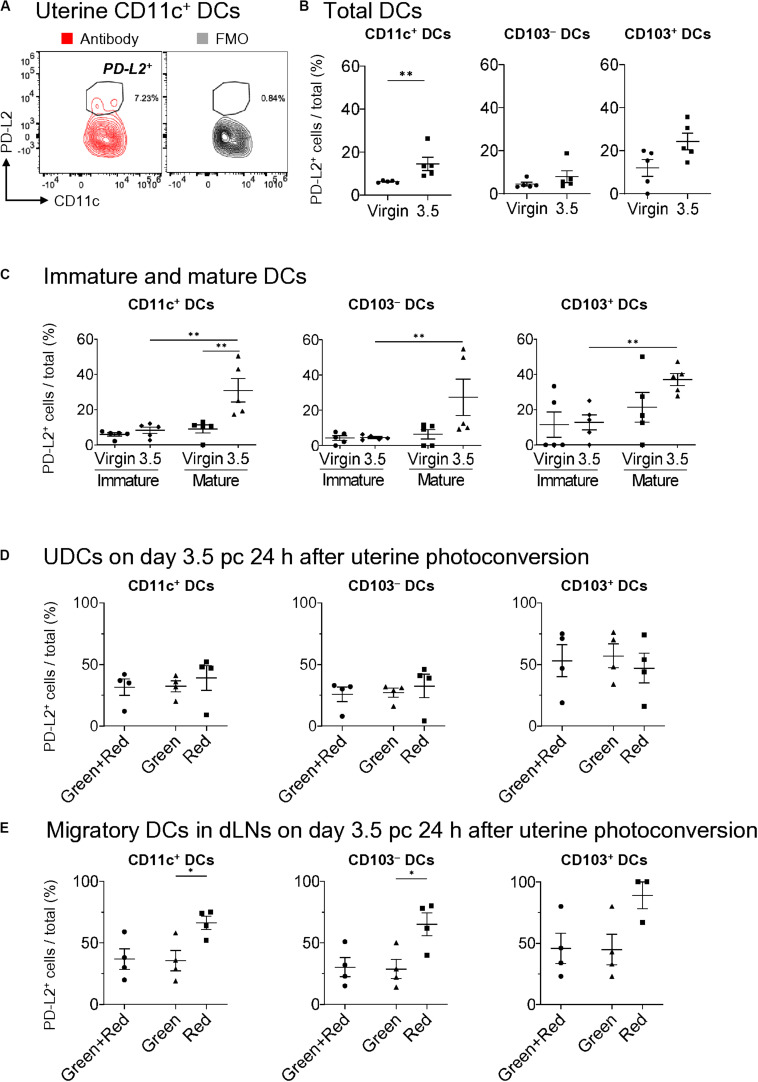
Expression of PD-L2 in uDCs increased immediately before implantation. **(A)** Flow cytometry contour plots show expressions of PD-L2 in uterine CD11c^+^ DCs with fluorescence minus one (FMO). **(B,C)** Proportion of PD-L2^+^ DCs of total DCs **(B)**, immature DCs, and mature DCs **(C)** in allogeneic mating at each time point. **(D,E)** Proportion of PD-L2^+^ DCs within the uterus **(D)** and dLNs **(E)** in allogeneic mating 24 h after uterine photoconversion on day 3.5 pc. A minimum of five **(B,C)** and four **(D,E)** samples from each time point were analyzed. Data represent mean ± SEM **(B,C)** and are representative of three independent experiments. Statistical comparisons were performed using Mann-Whitney *U*-test **(B,C)** and Kruskal-Wallis test with Dunn’s multiple comparisons test **(D,E)** (^∗∗^*P* < 0.01, ^∗^*P* < 0.05).

PD-L2 has been implicated to play a critical role in immune tolerance by negatively regulating the T cell immune response (27, 41). To clarify its contribution to tolerogenic conditioning, we analyzed PD-L2 expression on DCs (Figure 5A) and found that it was not expressed on mature and immature uDCs in virgin mice (Figures 5B,C). However, more than 30% of CD103^+^- and CD103^–^-mature DCs, but not immature DCs, expressed PD-L2 on day 3.5 pc in allogeneic mating (Figure 5C). Moreover, we examined results for day 3.5 pc—24 h after uterine photoconversion—to clarify the type of DCs expressing PD-L2. However, no clear differences were observed in PD-L2 expression between the migratory DCs and remaining DCs in the uterus on day 3.5 pc (Figure 5D). Meanwhile, a significant increase was observed in the expression of PD-L2 on uterus-derived CD11c^+^ DCs and CD103^–^ DCs in the uterine dLNs (Figure 5E), suggesting that the DCs expressing PD-L2 in uterine dLNs were actually migratory DCs from the uterus.

## Discussion

In this study, we showed that there is a transient increase in CD11c^+^ CD86^high^ MHC class II^high^ Ly6C^–^ PDCA-1^dim^ CD11b^+^ CD103^–^-mature uDCs on day 0.5 pc, and CD11c^+^ CD86^dim^ MHC class II^dim^ Ly6C^–^ PDCA-1^dim^ CD11b^–^ CD103^+^-immature uDCs on day 3.5 pc. The mature DCs were observed following allogeneic- and syngeneic mating on day 1.5 pc, suggesting that sexual intercourse itself, or semen, induced accumulation of mature DCs in the uterus after coitus. After seminal priming, semen stimulates uterine inflammation through the γδ T cells/IL-17A axis (35). Moderate inflammation is proposed to play an important role in successful implantation (32). Additionally, even mechanical injury of the endometrium can contribute to the receptivity of uterus for implantation with the accumulation of DCs (42, 43). It is proposed that moderate inflammation also contributes to the infiltration of DCs to the uterus, which induces feto-maternal tolerance by recognition of paternal antigens (32, 33, 44). Our study showed that paternal antigens did not affect the increase in uDCs on day 0.5 pc as the increased uDCs on day 1.5 pc were observed in both allogeneic- and syngeneic mating. Hence, the increase in mature uDCs might play a role in successful implantation and decidualization in allogeneic- and syngeneic mating. However, the role of inflammation in successful implantation remains unknown. Moreover, further investigations are required to confirm the role of uDCs on day 1.5 pc in the process of implantation.

The migration and pre-existence of uDCs using CFSE labeling in non-pregnant mice has been reported, however, little is known about the process by which resident and migratory uDCs contribute to successful implantation (1). Our study was the first to show turnover in the uterus and migration of uDCs to the dLNs using KikGR mice. We found that half of uDCs interchanged from day 0.5 to 1.5 pc. Interestingly, uterine remaining DCs were almost mature DCs, and an equal number of immature and mature made up the migratory DC population on day 1.5 pc. In general, infiltrating DCs—which are immature phenotype—migrate to peripheral organs and are subsequently matured. After coitus uDCs are almost matured for 24 h. Importantly, we revealed that uDCs before implantation consist of two types, namely immature DCs and CD103^–^-and CD103^+^-mature DCs expressing PD-L2. Immature DCs, which have been previously reported (1, 45), have low antigen presenting capacity. These immature uDCs regulate maternal T cell-activation against fetal antigens and induce paternal antigen-specific Tregs *in vitro* via seminal plasma (13). Our data showed that the frequency of immature uDCs was significantly higher in allogeneic mating as compared to that in syngeneic mating on day 3.5 pc, suggesting that immature uDCs may prevent rejection of the semi-allogenetic fetus by regulating the maternal immune system. Interestingly, our data showed that approximately 68% of uDCs were transformed to migratory DCs from day 2.5 to 3.5 pc. These migratory DCs may play important roles in successful implantation by regulating decidualization and angiogenesis, as these DCs regulate the activation of T cells (1, 2, 5–8). Additionally, we observed the presence of immature uDCs immediately before implantation originating from migratory DCs, however, it was unclear the origins of these immature DCs, which must be investigated in future. Similarly, the mechanism by which DCs migrate from the periphery to the uterus should also be characteraized.

Immature DCs are typically reported to be tDCs (1, 45), however, another type of uDCs found on day 3.5 pc were the CD103^–^- and CD103^+^-mature DCs expressing PD-L2 (Figures 5B,C). We showed that a large proportion of the uterus-remaining mature DCs express PD-L2 immediately before implantation. These PD-L2-expressing cells may stimulate effector T cells with an inhibitory signal via the PD-L2/PD1 pathway, thereby inhibiting their effector T cell functions allowing for successful implantation during allogeneic pregnancy. However, it has been reported that the number of paternal antigen-specific Tregs increase in the dLNs before implantation during allogeneic, but not syngeneic mating (13, 40). The DCs in the dLNs that migrated from the uterus were of the CD103^–^- and CD103^+^-mature phenotype, suggesting that they could take up the paternal allo-antigen from the semen and migrate to the dLNs, wherein they could effectively stimulate both the paternal allo-antigen-specific-CD4^+^, and -CD8^+^ effector T cells, as well as the Tregs in dLNs before implantation (23).

Although few reports have detailed DC subtypes in the uterus, we evaluated not only CD103^–^ DCs, CD103^+^ DCs, and pDCs in allogeneic- and syngeneic pregnancy, but also XCR1^+^ DCs and XCR1^–^ DCs in virgin mice. To our knowledge, this is the first study reporting CD103-expressing uDCs in cDC2s. Thus far, many reports on cDCs have shown cDC1s and cDC2s as CD103^+^ DCs and CD11b^+^ DCs, respectively (46–50) and, like the DC classification of intestines (26, 39, 51), it would be necessary to investigate the expressions of XCR1 and SIRPα before detecting the expression of CD103, although, the distribution of cDC1s and cDC2s in the uterus was not determined in our present study. Additionally, as a limitation of this study, the functional analysis of migratory DCs was not examined, thus, further examination will be required to determine the relative abundance of proliferation, activation markers, and functional analysis of the uterine cDC1s and cDC2s. During DC turnover analysis we did not examine uterine photoconversion from day 0 to 1.0 pc, as there was a limitation of natural sexual intercourse after photoconversion. In addition, as a topic for further study, we plan to examine the role of sperm and seminal plasma in the induction of DC differentiation. Further research is also required to investigate the induction of Tregs and anti-inflammatory cytokines, or tolerogenic functions of each DC subset, as well as the properties of human uDC subsets associated with implantation failure.

In conclusion, we comprehensively demonstrated the coitus-induced uDC dynamics associated with preparing tolerogenic conditions in the uterus and dLNs at the time of implantation. Importantly, we revealed that uDCs before implantation consist of two types of DCs: the immature DCs, as it has been previously proposed, as well as the CD103^–^- and CD103^+^-mature DCs expressing PD-L2, which may present paternal allo-antigens to CD4^+^ and CD8^+^ T cells with an inhibitory signal, thereby inhibiting their effector T cell functions. Our findings deepen the current understanding regarding the reproductive immune response before implantation, and may serve to provide new targets for the prevention of implantation failure.

## Materials and Methods

### Mice

C57BL/6 and BALB/c mice were purchased from CLEA Japan. Knock-in mice carrying KikGR cDNA under the CAG promoter (KikGR mice) were generated as previously described (36, 37). Mice were bred and maintained in a specific pathogen-free facility at Osaka Ohtani University. All animal procedures were performed in accordance with the institutional guidelines of the Animal Research Committee of Osaka Ohtani University. C57BL/6 female mice aged 8–10 weeks were mated with BALB/c or C57BL/6 male mice. KikGR knock-in C57BL/6 female mice aged 8–10 weeks were mated with BALB/c male mice. The presence of vaginal plugs was determined the next morning and females were then separated from the males. The presence of vaginal plugs marked day 0.5 of pregnancy. Pregnant mice were euthanized on days 0.5, 1.5, 2.5, and 3.5 pc. Six days before photoconversion, non-pregnant virgin mice were synchronized in the diestrous stage via subcutaneous injection with 2 mg medroxyprogesterone (MPA) (Tokyo Chemical Industry, Osaka, Japan).

### Photoconversion

During photoconversion of the uterus, non-photoconverted regions were protected from light using aluminum foil, while the region of the uterus targeted for photoconversion was exposed to violet light (405 nm, 100 mW/cm^2^) for 2 min from the front and behind following laparotomy. Following photoconversion, the abdominal wall was closed. To keep the exposed tissues moist during exposure to light, warmed PBS was applied to the region of photoconversion. To prevent hypothermia, mice were warmed with a heater during the perioperative stages.

### Reagents, Antibodies, and Flow Cytometric Analysis

Mononuclear cells were isolated from dLNs and the uterus on days 0.5, 1.5, 2.5, and 3.5 pc. Resected uteri were minced with scissors, and the tissues were then passed through a 100-μm cell strainer. The antibodies used were as follows: purchased from BD, eBioScience, or BioLegend: FITC-conjugated anti-I-A/I-E (clone M5/114.15.2), plycoerythrin (PE)-conjugated anti-CD103 (clone 2E7), PE-Dazzle594-conjugated anti-CD11c (clone N418), PE-cyanine5-conjugated anti-CD45R/B220 and streptavidin (clone RA3-6B2), PE-cyanine7-conjugated anti-Gr-1 and I-A/I-E (clone RB-6-8C5, and M5/114.15.2, respectively), allophycocyanin (APC)-conjugated anti-PDCA-1, CD26,and SIRPα (clone 927, H194-112, and P84, respectively), Alexa Flour 700-conjugated anti-CD45 (clone 30-F11), APC-cyanine7-conjugated anti-F4/80 and CD45 (clone BM8 and 30-F11, respectively), APC-R700-conjugated anti-CD103 (clone M290), Brilliant Violet (BV) 421-conjugated anti-CD86, CD64, and XCR1 (clone GL-1, X54-5/7.1, and ZET, respectively), Pacific blue-conjugated anti-CD11b (clone M1/70), BV510-conjugated anti-Ly6C, CD45, and CD11c (clone HK1.4, 30-F11, and N418, respectively), and biotin-conjugated anti-CD273 (clone TY25). For flow cytometric analysis, cells were washed with Dulbecco’s PBS supplemented with 2% fetal calf serum (FCS), and 0.02% sodium azide. Next, cells were incubated with 2.4G2 hybridoma culture supernatant to block Fc binding. Dead cells were labeled with PI. Stained samples were acquired using SP6800 (SONY, Tokyo, Japan). KikGR-green and red signals were detected using 530/60 and 595/50 bandpass filters, respectively. Flow cytometry data were analyzed using the FlowJo software (Tree Star, Ashland, OR, United States).

### Data Analysis

Dimensionality reduction was performed using tSNE analysis, followed by FlowJo. First, we exported PI^–^ CD45^+^ Gr-1^–^ F4/80^–^ CD11c^+^ MHC class II^+^ B220^–^ (CD11c^+^ DCs) and PI^–^ CD45^+^ Gr-1^–^ F4/80^–^ CD11c^+^ PDCA-1^+^ CD11b^–^ Ly6C^+^ B220^+^ (pDCs) compartments from each dataset, and the cell numbers were adjusted to be the same as those in the minimal sample for each gestational age, including a minimum of five uterine samples. Next, the data was concatenated and visualized as a two-dimensional map by tSNE.

Mann-Whitney *U*-test and Kruskal-Wallis test with Dunn’s multiple comparisons test were performed using GraphPad Prism version 8.4.3 (GraphPad Software, San Diego, CA, United States). Data in bar graphs represent mean ± standard error of mean (SEM). *P*-values <0.05 were considered to be statistically significant.

## Data Availability Statement

The raw data supporting the conclusions of this article will be made available by the authors, without undue reservation.

## Ethics Statement

The animal study was reviewed and approved by Osaka Ohtani University.

## Author Contributions

IY designed the study, performed the experiments, analyzed the data, and wrote the manuscript. TM, RI, YK, AU, TS, and AN performed the experiments and analyzed the data. MT, TS, AN, and SS designed the study and wrote the manuscript. All authors read and approved the final version of the manuscript.

## Conflict of Interest

The authors declare that the research was conducted in the absence of any commercial or financial relationships that could be construed as a potential conflict of interest.

## Abbreviations

cDCs: conventional DCs
DCs: dendritic cells
dLN: draining lymph node
KikGR: Kikume Green Red
LNDCs: lymph node DCs
MPA: medroxyprogesterone
pc: post coitus
pDCs: plasmacytoid DCs
PI: propidium iodide
tDCs: tolerogenic DCs
Tregs: regulatory T cells
tSNE: t-distributed stochastic neighbor embedding
uDCs: uterine dendritic cells.
