# Supplementary material for: Dynamic Changes in the Phenotype of Dendritic Cells in the Uterus and Uterine Draining Lymph Nodes After Coitus
Source: Front Immunol. 2020 Sep 11;11:557720. doi: 10.3389/fimmu.2020.557720 (PMC7516021; doi:10.3389/fimmu.2020.557720)
Supplement: Supplementary file 1 [file Data_Sheet_1.PDF]

Supplementary Material

Supplementary Figures

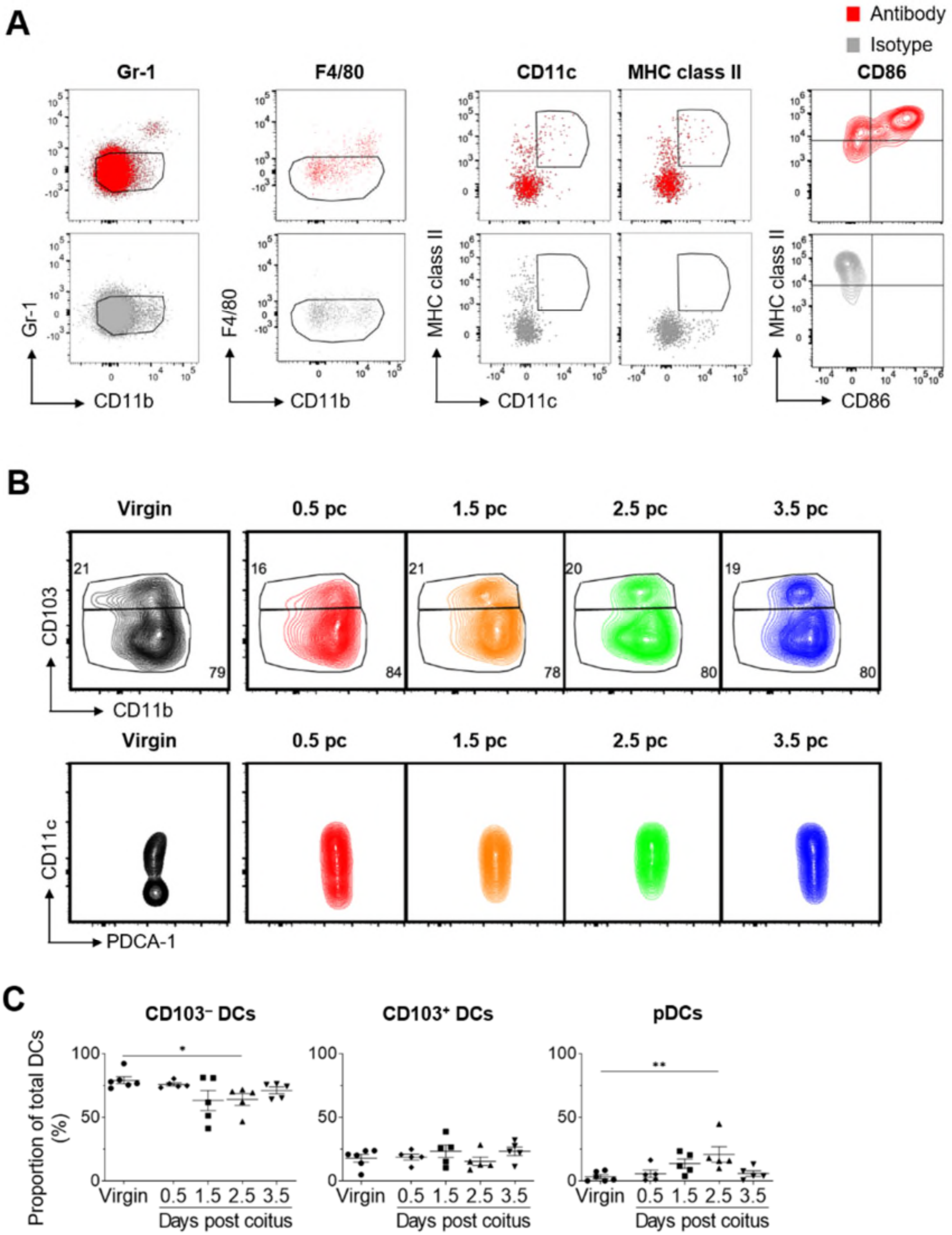

Supplementary Figure 1. Proportion of uDC subset after coitus in allogeneic mating

(A) Flow cytometry dot, and contour plots showing the fluorescence intensity of each antibody isotype. (B) Flow cytometry contour plots showing representative plots of CD103^{-} DCs, CD103^{+} DCs, and pDCs within the uterus at each time point. (C) Time course in proportion to each DC subset. At minimum of five samples from each time point were analyzed. Data represent mean ± SEM and are representative of three independent experiments. Statistical comparisons were performed using the Kruskal-Wallis test with Dunn's multiple comparisons test.

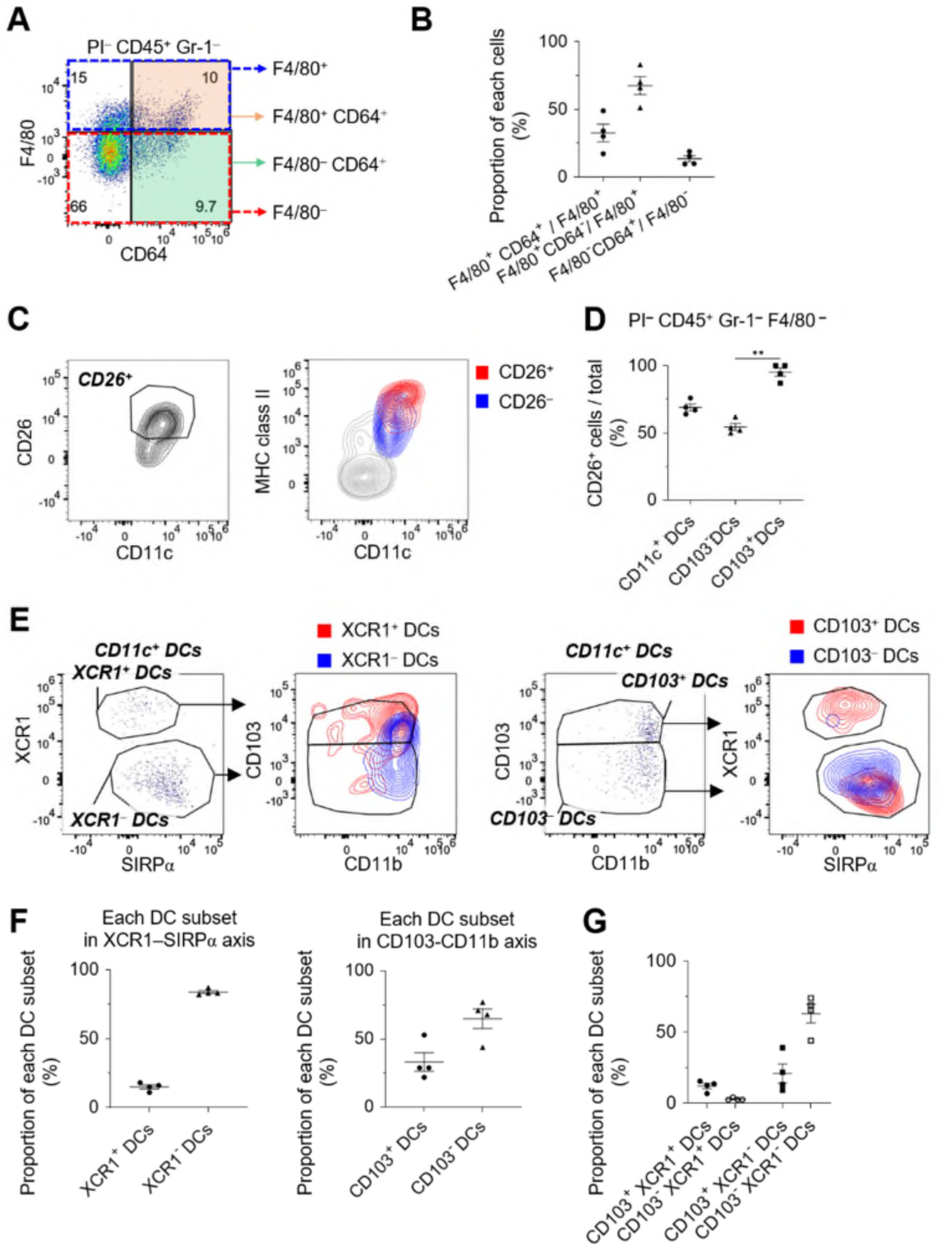

Supplementary Figure 3. UDC classification via expression of CD64, CD26, XCR1, and SIRPα

(A) Relative expression of F4/80 and CD64 in PI⁻ CD45⁺ Gr-1⁻ cells. (Upper (Blue): F4/80⁺, right upper (orange): F4/80⁺ CD64⁺, right lower (green): F4/80⁻ CD64⁺, lower (red): CD64⁺). (B) Proportion of CD64⁻ and CD64⁺ cells among F4/80⁺ cells (left), and CD64⁺ cells among F4/80⁻ cells (right). (C) Relative expression of CD26 in uterine CD11c⁺ DCs (left), and comparative expression of CD11c and MHC class II between CD26⁺ and CD26⁻ cells (right). (D) Proportion of CD26⁺ DCs in each DC subset. (E) uDC expression in XCR1/SIRPα (left) and CD103/CD11b gating (right). (F) Proportion of each DC subset in XCR1/SIRPα, and CD103/CD11b gating strategy. (G) Proportion of each DC subset based on the expressions of CD103 and XCR1. A minimum of four samples from virgin mice were analyzed. Data represent mean ± SEM and are representative of three independent experiments. Statistical comparisons were performed using the Kruskal-Wallis test with Dunn's multiple comparisons test (D) and (G). (**$P < 0.01$, *$P < 0.05$)

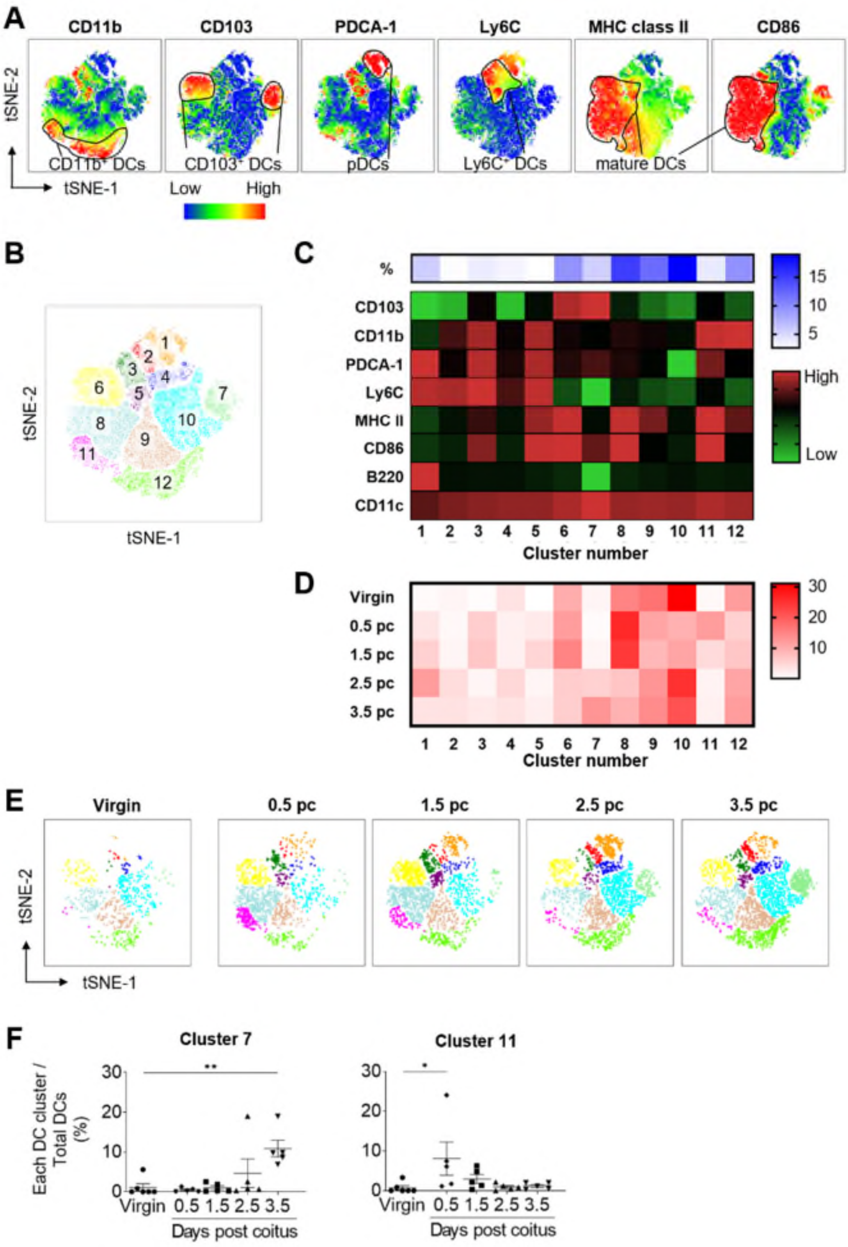

Supplementary Figure 3. Time course for uDCs in allogeneic mating by t-distributed stochastic neighbor embedding analysis

T-distributed stochastic neighbor embedding (tSNE) projection for DC diversity from pooled data of individual CD11c$^+$ DCs and pDCs within the uterus across each time point (n = 26). (A) tSNE map shows the expression of DC molecules. (B) Clusters of tSNE map were manually generated by analyzing the expression of DC molecules and the time course of cluster changes. (C and D) Heatmaps show the proportion, DC molecule, and time course of proportion changes of each clusters. Scale of DC molecule (C) show $\log_{10}$ MFI. (E) tSNE map shows the time course of cluster changes. (F) Proportion of each cluster at each time point. A minimum of five samples from each time point were analyzed. Data represent mean ± SEM (F) and are representative of three independent experiments. Statistical comparisons were performed using the Kruskal-Wallis test with Dunn's multiple comparisons test. (**P < 0.01, *P < 0.05)

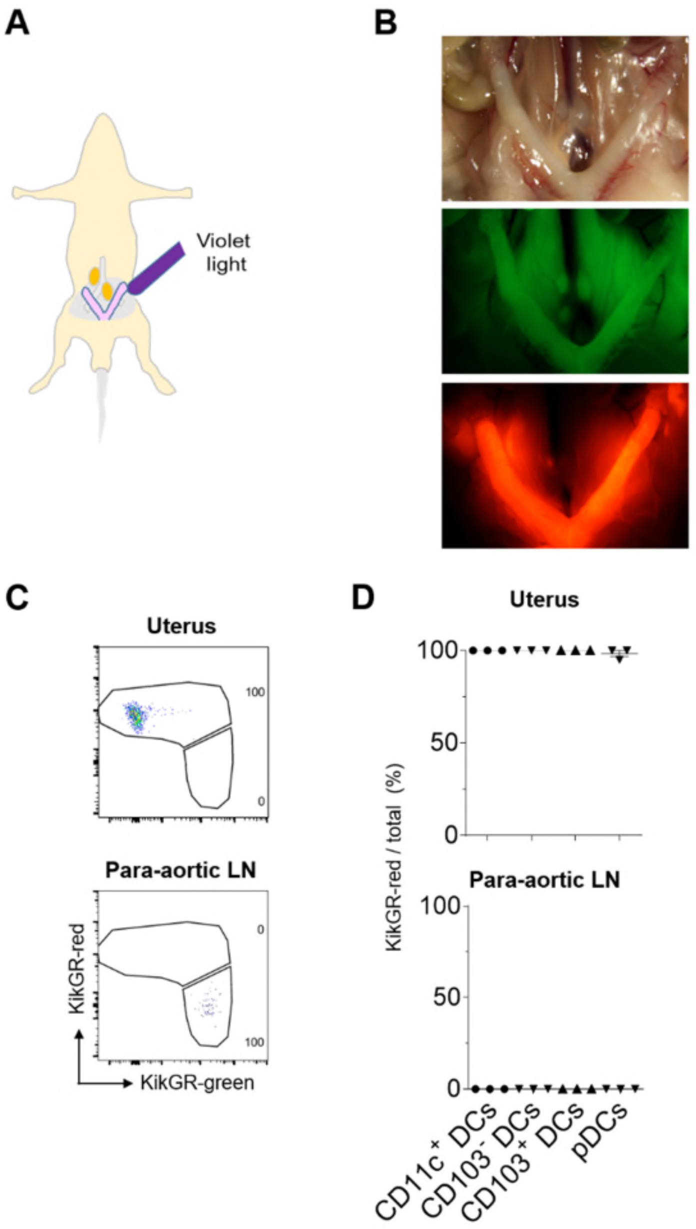

Supplementary Figure 4. Monitoring DC movement in the uterus using the photoconvertible protein KikGR mice

(A and B) KikGR mice were irradiated by violet light with longitudinal incision and photoconversion of the KikGR protein was induced from green (KikGR-green) to red (KikGR-red). (C) The uterus and draining para-aortic LNs were resected immediately after photoconversion, and the cells from these tissues were isolated for flow cytometry analysis. Flow cytometry plots show KikGR-green and KikGR-red DCs ($CD11c^+$ DCs) within the uterus and dLNs just after photoconversion. (D) Total uterine DCs were further gated into $CD103^-$ DCs, $CD103^+$ DCs, and pDCs. Graph shows the proportion of each DC subset labeled KikGR-red just after photoconversion. A minimum of three samples from each time point were analyzed. Data represent mean ± SEM (C and D) and are representative of three independent experiments. Statistical comparisons were performed using the Kruskal-Wallis test with Dunn's multiple comparisons test.

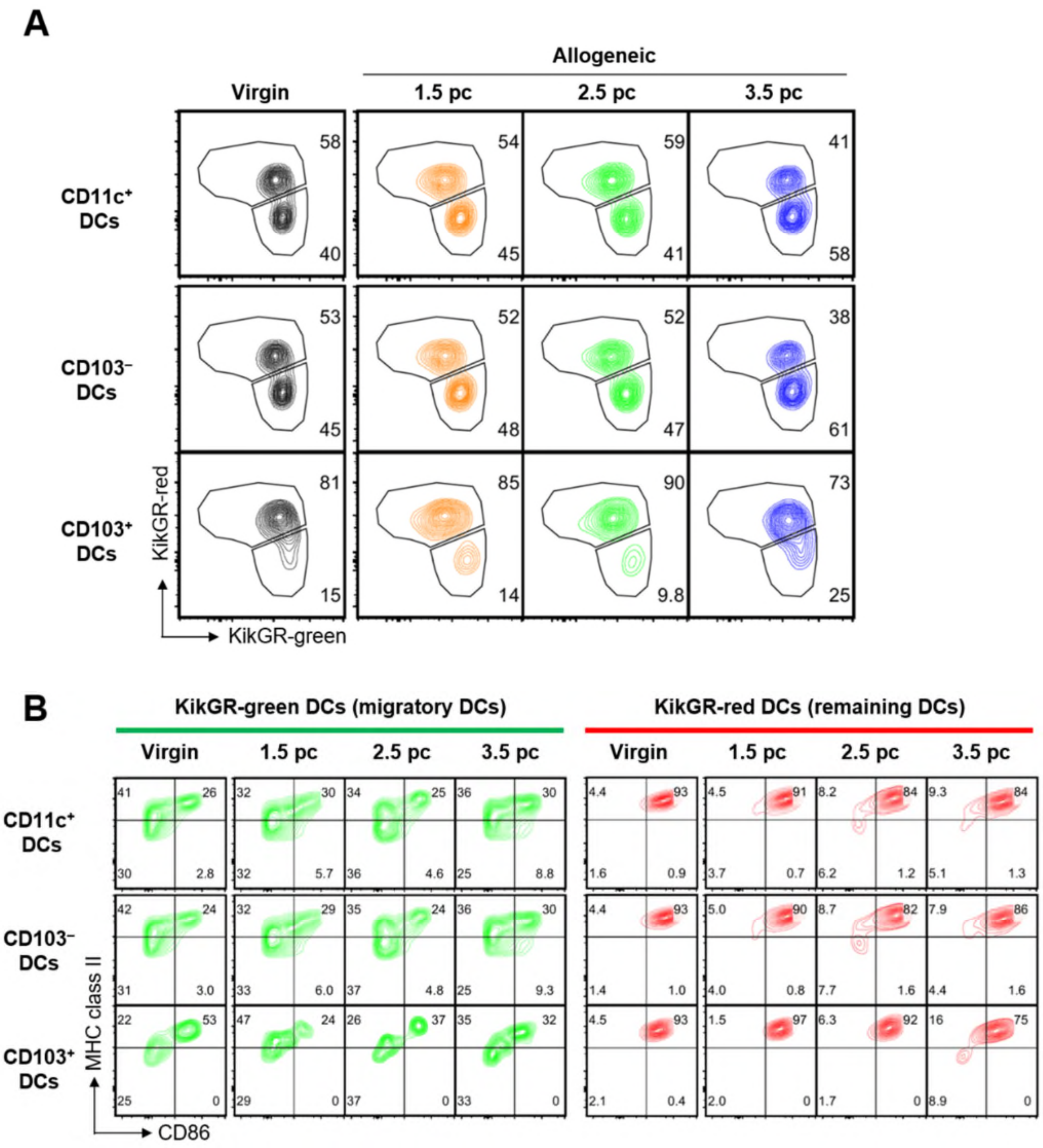

Supplementary Figure 5. Time course for migratory DCs and remaining DCs in the uterus after coitus in allogeneic mating

(A and B) Proportion of KikGR-green and KikGR-red cells (A) and proportion of immature and mature DCs of KikGR-green and KikGR-red cells (B) within each DC subset in the uterus 24 h after photoconversion at each time point (B). A minimum of three samples from each time point were analyzed.

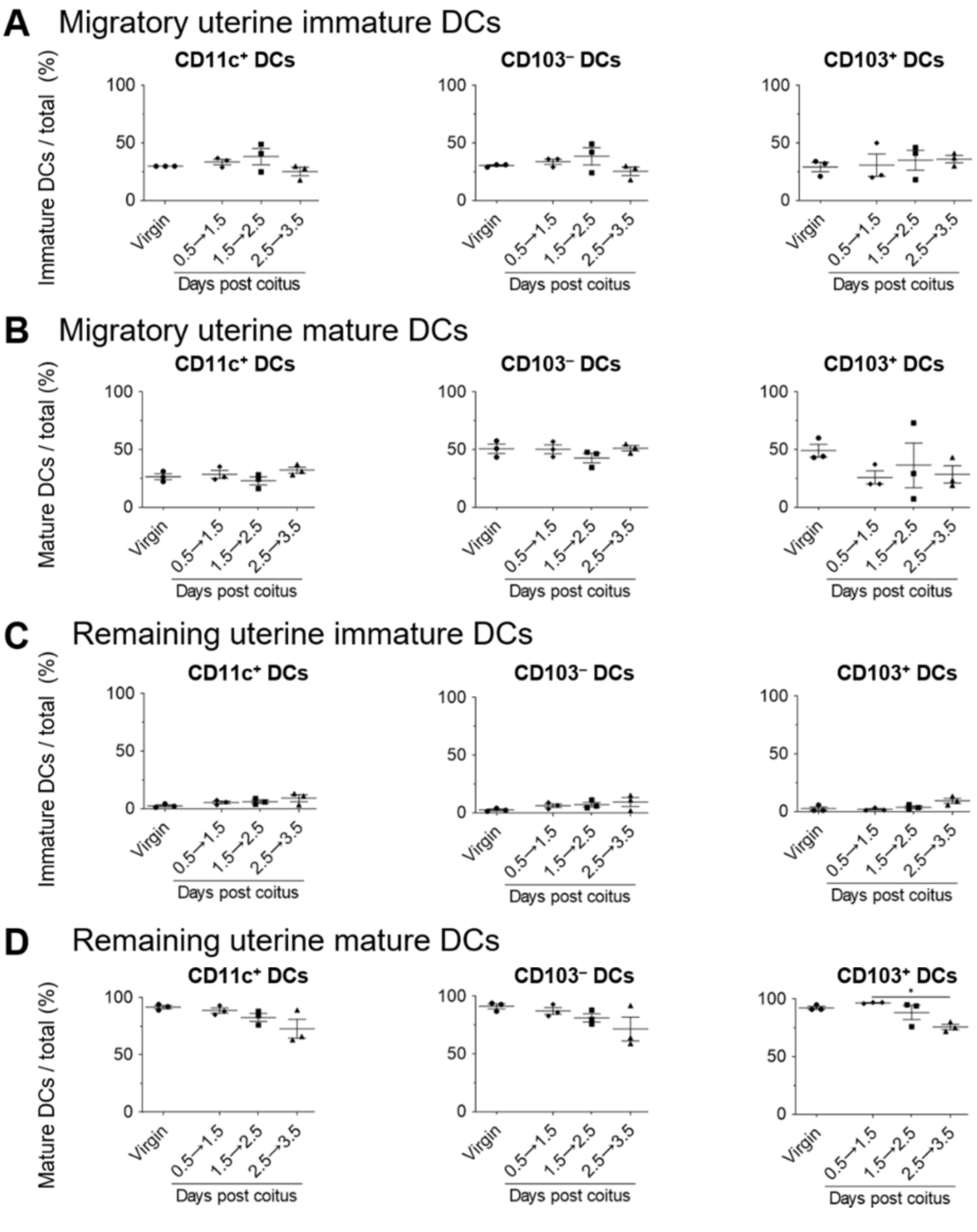

Supplementary Figure 6. Proportion of immature- and mature DCs in migratory DCs and remaining DCs 24 h after photoconversion

(A-D) Relative proportion of immature DCs and mature DCs of the total DCs in each DC subset labeled with KikGR-green (A and B) and KikGR-red (C and D). A minimum of three samples were analyzed. Data represent mean ± SEM (C) and are representative of three independent experiments. Statistical comparisons were performed using the Kruskal-Wallis test with Dunn's multiple comparisons test. (*P < 0.05)

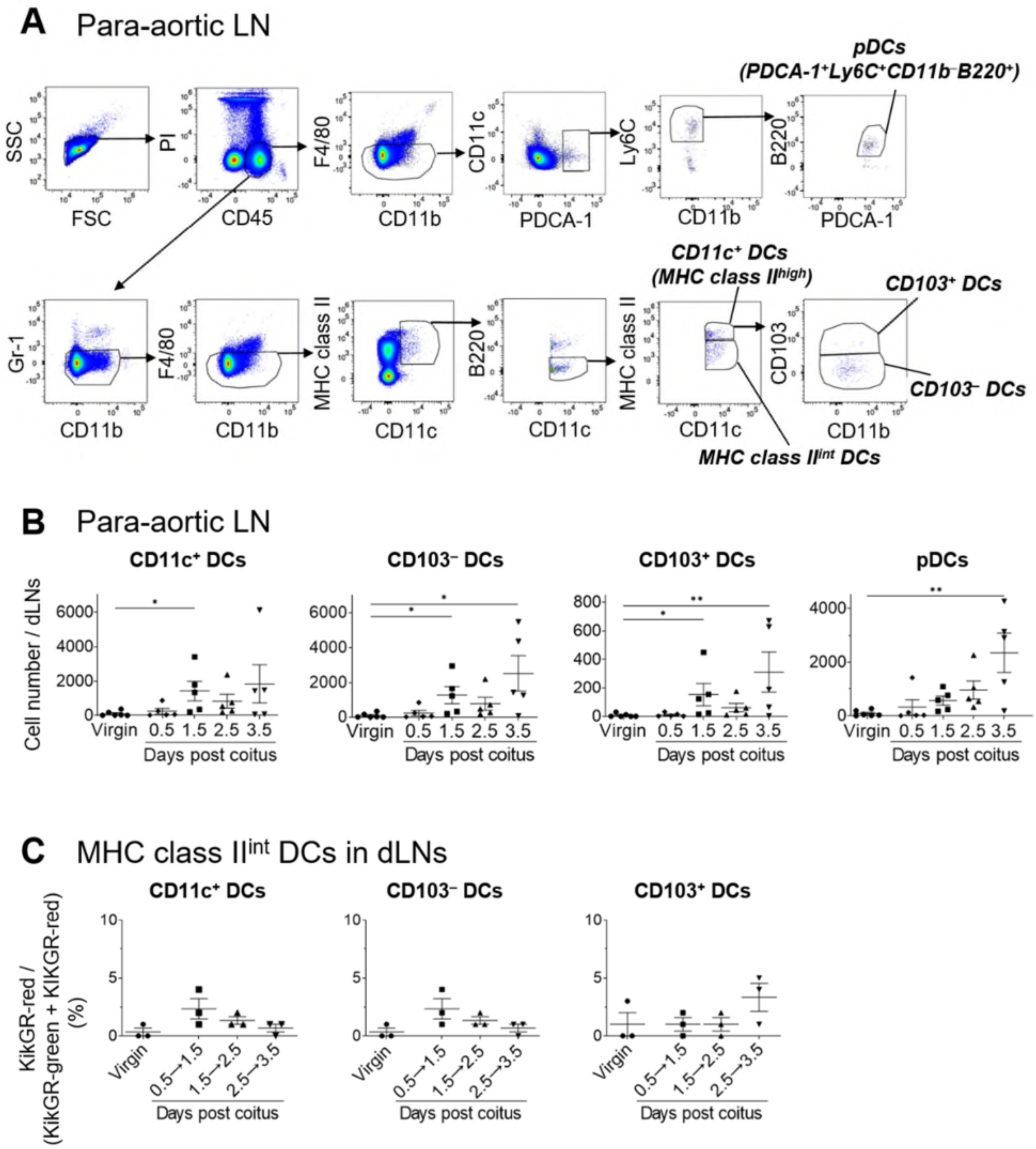

Supplementary Figure 7. Number of migratory DCs within para-aortic lymph nodes in allogeneic mating

(A) Gating strategy used to identify $CD103^{-}$ DCs, $CD103^{+}$ DCs, and pDCs in the draining para-aortic lymph nodes. (B) Total number in each DC subset at each time point. (C) Proportion of each DC subset labeled KikGR-red out of MHC class II^{int} DC subset 24 h after photoconversion in dLNs. A minimum of three samples from each time point were analyzed. Data represent mean ± SEM (B and C) and are representative of three independent experiments. Statistical comparisons were performed using the Kruskal-Wallis test with Dunn's multiple comparisons test. (**$P < 0.01$, *$P < 0.05$)
